# Aphid Gel Saliva: Sheath Structure, Protein Composition and Secretory Dependence on Stylet-Tip Milieu

**DOI:** 10.1371/journal.pone.0046903

**Published:** 2012-10-02

**Authors:** Torsten Will, Kathrin Steckbauer, Martin Hardt, Aart J. E. van Bel

**Affiliations:** 1 Plant Cell Biology Research Group, Department of General Botany, Justus-Liebig-University, Giessen, Germany; 2 Central Biotechnical Facility, Justus-Liebig-University, Giessen, Germany; Institute of Molecular and Cell Biology, Singapore

## Abstract

In order to separate and analyze saliva types secreted during stylet propagation and feeding, aphids were fed on artificial diets. Gel saliva was deposited as chains of droplets onto Parafilm membranes covering the diets into which watery saliva was secreted. Saliva compounds collected from the diet fluid were separated by SDS-PAGE, while non-soluble gel saliva deposits were processed in a novel manner prior to protein separation by SDS-PAGE. Soluble (watery saliva) and non-soluble (gel saliva) protein fractions were significantly different. To test the effect of the stylet milieu on saliva secretion, aphids were fed on various diets. Hardening of gel saliva is strongly oxygen-dependent, probably owing to formation of sulfide bridges by oxidation of sulphydryl groups. Surface texture of gel saliva deposits is less pronounced under low-oxygen conditions and disappears in dithiothreitol containing diet. Using diets mimicking sieve-element sap and cell-wall fluid respectively showed that the soluble protein fraction was almost exclusively secreted in sieve elements while non-soluble fraction was preferentially secreted at cell wall conditions. This indicates that aphids are able to adapt salivary secretion in dependence of the stylet milieu.

## Introduction

Aphids feed from sieve tubes, channels for transport of organic nutrients in higher plants. Prior to sieve-tube feeding, an aphid stylet makes its way towards the phloem and is probably guided by plant cues [1], [2]. As the stylet advances through the plant tissue, the aphid secretes gel saliva that hardens soon after secretion and forms a continuous sheath encasing the full length of the stylet inside the plant. The stylet pathway prior to reaching a sieve tube is primarily intercellular. Along this path, cells are periodically probed and injected with a minute amount of watery saliva [3], [4], which is produced by the accessory saliva glands [5]. When the stylet finally punctures a sieve element (SE), representing a single module of a sieve tube, large amounts of watery saliva are secreted into it. After this initial salivation phase, aphids start ingesting SE sap while watery saliva is intermittently secreted and mixes with the ingested SE sap [6].

As one principal function, watery saliva appears to counteract plant defense reactions against aphid feeding (e.g. [7], [8], [9]). For example, Ca^2+^ influx into SEs causes sieve-tube occlusion by proteins and callose [10]. Ca^2+^ influx accompanying stylet puncture [11] would therefore bring about a stop of mass flow [12]–[14], hence cutting off the food supply of aphids. Calcium-binding proteins in watery saliva [7], [9] are likely to suppress Ca^2+^-triggered sieve-tube occlusion [7], [11]. Apart from the ability to sabotage sieve-tube occlusion, which is probably widespread among aphids [15], several other functions of watery-saliva proteins (e.g., as effectors inside the plant) have been postulated [16]. Their tasks may be diverse, since the protein composition of watery saliva seems to widely vary among aphid species [15].

By contrast, virtually nothing is known about the protein composition and functions of gel saliva [17]. Gel saliva is presumably produced by the principal salivary glands [5], [18]. The first event in gel saliva secretion is the secretion of a small amount (the salivary flange) on the plant surface which provides a holdfast for initiation of stylet penetration into the plant [6], [19]. Once the stylet penetrates, it proceeds through cell walls [20], possibly along the weak middle lamellae. Gel saliva is continuously secreted during stylet movement through the plant cortex towards the phloem, and it rapidly hardens to form a continuous sheath around the stylet [21]. The sheath is formed stepwise by repeated secretion of minuscule drops of gel saliva; the stylet then advances through the newly hardened drop of sheath saliva and the next pulse of gel saliva is secreted and adheres to the previous [22]. Traces of gel saliva, secreted during probing of Parafilm-covered artificial diet, show the successive drops as a “pearl necklace” like structure attached to Parafilm [23], [24].

Gel-saliva proteins may “polymerize” by oxidation of sulphydryl-groups that leads to sheath hardening [6], [24]. Miles [25] showed that sheath material is able to detoxify phenolic compounds *in vitro*, probably by oxidases and free sulphydryl-groups of proteins. Gel saliva is in addition regarded as a lubricating substance to facilitate stylet advancement and retraction; it may also protect the stylet from mechanical damage and isolate it from chemical attacks (e.g., by plant chitinases [26]).

To the best of our knowledge, only one study on the protein composition of gel saliva has been published [18]. In our work, we examined the sheath structure by confocal laser scanning microscopy (CLSM) and scanning electron microscopy (SEM). Additionally, we analyzed the protein content of gel saliva after collection of gel saliva sheaths deposited onto Parafilm membranes, by using a novel solubilization approach. Protein composition of gel and watery saliva secreted into artificial diets under diverse diet conditions were compared after SDS-PAGE separation.

## Materials and Methods

### Aphid and plant breeding

The aphid species *Megoura viciae* (Buckton) was cultured on two- to three-week old *Vicia faba* cv. Witkiem major plants in Perspex® cages with large gauze-covered windows. The aphid species *Macrosiphum euphorbiae* was reared on four- to five week old *Cucurbita maxima* plants without Perspex® cages. Conditions in a controlled environment room were set to a L17∶D7 (Light∶Dark) regime at an average temperature of 21°C. Plants used for aphid rearing were cultivated in a greenhouse with an average temperature of 20°C and natural light plus additional illumination (SONT Agro 400 W, Phillips, Eindhoven, The Netherlands) – under a L14:D10 regime. Both aphid species were used for experiments where solubilization of salivary sheaths was tested (Fig. S1). *M. euphorbiae* was used for salivary flange observations by scanning electron microscopy because the aphid remained on the leaf during SEM preparation protocol. All other experiments were conducted with *M. viciae,* which was used in a previous study [7].

### Saliva collection chambers

Aphids were collected from the plants and their number was determined as described previously [7]. 1,500–2,000 aphids were poured onto the upper surface of the Parafilm cover of saliva-collection chambers filled with artificial diets of diverse composition [7]. The collection chamber is a Perspex block into which a shallow bath of 9 cm radius and a depth of 1 mm had been milled. Sterilized collection chambers were filled with 3 ml of diet under sterile conditions. Aphids penetrated the Parafilm membrane with their stylets in order to reach the diet. Insoluble saliva compounds deposited on the Parafilm cover and soluble compounds secreted into the diet were recovered separately after 24 hours of foraging under environmental conditions equal to the aphid breeding conditions.

### Diets for aphid saliva collection

The type of saliva secreted is likely to differ among different phases of the stylet penetration/feeding process. Consequently, two basic diets were used for collection of aphid watery and gel saliva, which mimicked conditions in i) sieve-elements (SE) sap (15% sucrose, 100 mM L-serine, 100 mM L-methionine and 100 mM L-aspartic acid with a pH of 7.2 (KOH) [7], [18]) and ii) cell-wall (CW) milieu (20 mM KCl, 1 mM CaCl_2_, 10 mM MES, adjusted to pH 5.5 (KOH) [27]). Before use, diets were filtered sterile through Rotilabo® syringe filters (Carl Roth GmbH, Karlsruhe, Germany) with a PVDF membrane having a pore size of 0.45 μm.

### Confocal laser scanning microscopy

Salivary sheaths adhering to the lower side of the Parafilm membrane that had been exposed to the diet solution, were examined by confocal laser scanning microscopy (Leica TCS 4D, Leica Microsystems, Heidelberg, Germany) with a 40× water immersion objective.

### Scanning electron microscopy

To obtain images of salivary flanges, leaf sections of 5×5 mm, previously colonized with *M. euphorbiae* in clip cages for 7 days, were dehydrated in graded ethanol concentrations 60; 80; 90 and 95%) and, finally, in acetone, which was mixed with silica gel to withdraw water. Leaf pieces were attached on scanning electron microscopy specimen holders with carbon glue.

To determine the effect of oxidation on formation of salivary sheath structure, salivary sheaths of *M. viciae* were collected with three variations of ST diet: 1) diet outgassed under an argon atmosphere prior to feeding; 2). outgassed diet with addition of 1 mM dithiothreitol (DTT) to prevent oxidation of disulfide-bonds; and 3) non-treated diet. DTT was added to ST diet and not to CW diet because of the absence of reducing capacity below pH 7. Salivary sheaths were localized on the Parafilm membranes using a bright-field microscope with a 40× water immersion objective, excised, placed on scanning electron microscopy holders, and dried for three days in a desiccator with silica gel under vacuum. Samples were gold-sputtered and observed with a scanning electron microscope (Philips XL20, Royal Philips Electronics, Nijmegen, The Netherlands) at 10 kV.

### Solubilisation of salivary sheaths and sample preparation for gel electrophoresis

Parafilm membranes that had covered the feeding chambers were drawn over plastic Petri dishes with the diet exposed side upwards. Membrane surfaces were rinsed for 5 minutes with 3 ml of Millipore water to remove remnants of watery saliva and diet. Sheath material that adhered to the Parafilm [24] was denaturized and solubilized with 3 ml of a solution of 9 M urea, 4% CHAPS, 0.1% sodiumdodecylsulfate (SDS) and 2% DTT in Millipore water by gentle shaking on an orbital shaker at room temperature for 45 min. Degradation of sheath material by this solution was observed under a microscope with a 40× water immersion objective and photographed with a Canon PowerShot A85 (Fig S1). The degradation solution for microscope observations was mixed gently with a pipette every 20 min to simulate the above-mentioned shaking of larger samples. As controls, saliva sheaths were treated with Millipore water.

Following solubilization of the salivary sheaths, the denaturated gel saliva was concentrated, desalted and freed from detergents by precipitation. Each sample was mixed 1∶1 with 0.6 M trichloroacetic acid and incubated on ice for 10 min. The protein pellets collected after centrifugation at 20.000*g at 4°C for 10 min were washed with 100 µl cold acetone after the supernatant was removed and centrifuged again for 10 min. After discarding the supernatant, pellets were resuspended in a volume of 1 µl per 100 aphids in 1× reducing sample buffer (4× Roti-Load®1, Carl Roth GmbH, Karlsruhe, Germany diluted 1 3 with Millipore water) and were pooled. Saliva aliquots of approx. 2000 aphids each were stored at −80°C.

### One-dimensional SDS-PAGE of gel and watery saliva

Samples of watery saliva were prepared according to Will et al. [7]. Proteins in watery and gel saliva samples were separated by 1D SDS-PAGE [28] using a 4% stacking gel and a 10% separation gel in a Mini-PROTEAN^TM^ 3 electrophoresis cell (Bio-Rad Laboratories, Hercules, CA, USA). Precision Plus Protein^TM^ Standard – All Blue (Bio-Rad) was used as a protein marker. Gels were silver-stained [29], [30] and documented with a Gel Doc XR (Bio-Rad). Analysis was performed by Quantity One 1-D analysis software (Bio-Rad). Lane comparison data were plotted with SigmaPlot 8.0 (SPSS, Chicago, IL, USA).

### Reducing ability of watery saliva

To test watery saliva for potential reducing ability that could interact with oxidation mediated sheath hardening in an artificial diet environment, saliva of 16500 aphids, collected in SE diet, and concentrated by ultrafiltration (Vivaspin 20 and 2 with a 3,000 molecular weight (MW) cut-off and polyethersulfon membrane; Sartorius, Goettingen, Germany) from a starting volume of approximately 35 ml to a final volume of 165 µl, was used. A similar amount of Millipore water was used as a control. The respective samples were mixed with 1.5 ml of a 0.0005 M methylene blue solution, prepared with ST diet. Methylene blue becomes colorless when reduced. Samples were incubated at 35°C for 72 h and extinction was measured at 668 nm.

### Image analysis and artwork

Size measurements in microscopic images were done with ImageJ 1.42q (Wayne Rosband, National Institute of Health, USA). Quality of photographs and microscopic images was improved for brightness and contrast using Corel® Photo-Paint 10 (Corel Corp. Ltd., Ottawa, Canada) after their respective analyses. Images that are part of an image set were treated equally.

## Results

### Salivary flange during and after stylet penetration

**Figure 1:**
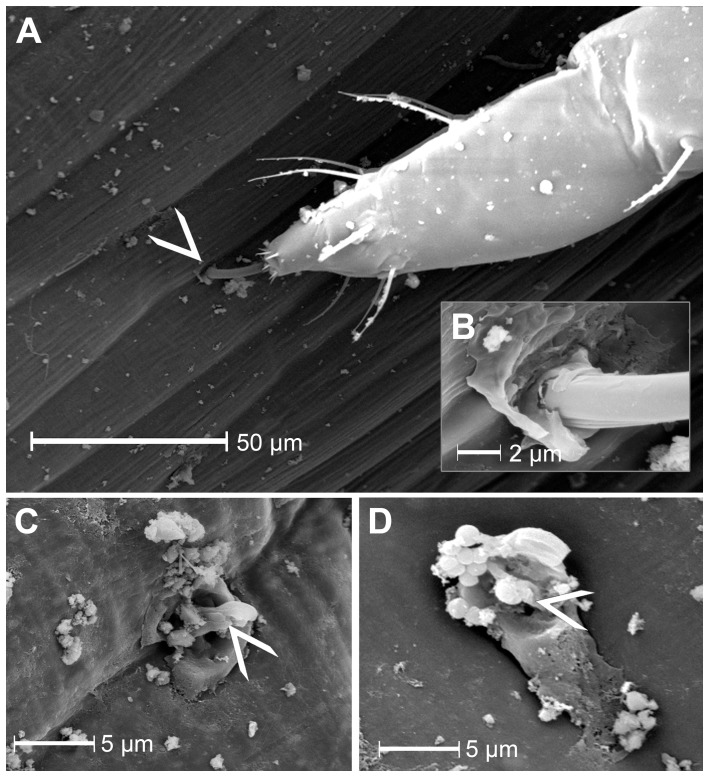
Scanning electron microscopy of stylet penetration sites of *M. euphorbiae*. **A**) The stylet entry point (arrowhead), which always seems to be located at the subsiding rims of anticlinal wall junctions between epidermal cells (N = 5), **B**) (magnified detail from **A**) is surrounded by a salivary flange. The saliva material partially covers the leaf surface around the stylet. **C**) After stylet retraction, the salivary flange appears to be plugged (N = 10). In the centre of the salivary sheath (arrowhead) saliva material shows a small elevation. Bacteria, spherical structures in the upper left region of the flange, reside on the gel saliva material. Growth of bacteria on the salivary flange material was observed regularly.

Gel saliva is secreted at the stylet penetration site that is located at the wall junction of two epidermal cells and forms a flange that surrounds the stylet tip (Fig. 1A, B). After stylet retraction, salivary flanges remain on the leaf surface and appear to be plugged by gel saliva material (N = 10), because a stylet entry point cannot be observed (Fig. 1C, D). Sometimes gel saliva is seen as elevated material (marked with arrow head; Fig 1C, d) while in other cases it appears as a smear (not shown).

### Saliva sheath formation and influence of oxygen

**Figure 2:**
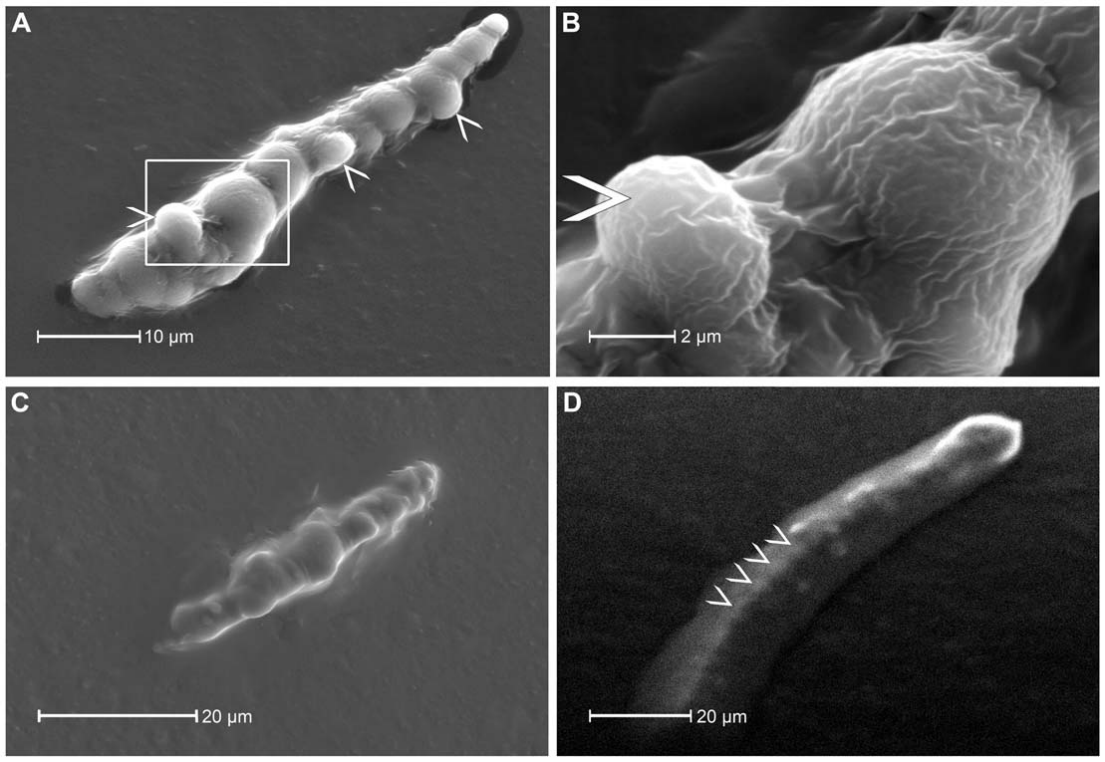
SEM micrographs of salivary sheaths from the aphid *M. viciae* formed under different oxidizing conditions. Sheaths in each micrograph are entirely attached to the Parafilm membrane that covered the diet; the initial stylet penetration site in Fig. **2A**, **C** and d is located in the lower left corner. **A**) A salivary sheath, formed under regular oxygen conditions, shows the typical pearl-necklace structure. The spherical parts of the gel saliva are formed by individual pulses of gel saliva and show clear delimitation from each other. The salivary sheath with a total length of approximately 52 µm tapers towards the tip and shows three short branches (arrowheads) in nearly regular intervals of 12.2; 16.9 and 13.1 µm (mean: 14.07 µm). The area within the white frame is zoomed (**B**) and shows a rough texture of the salivary sheath. A short branch is marked with an arrow head. **C**) A gel saliva sheath formed under oxygen-reduced conditions with less clear delimitation of the single saliva drops and a smoother texture than under oxygen conditions. The sheath structure appears to be more flat than under oxygen conditions. **D**) Addition of 1 mM DTT to low-oxygen ST-diet leads to a complete loss of the sheath structure. Potential remnants of the pearl-necklet appearance (shown exemplarily by white arrowheads) are visible at regular intervals inside the amorphous sheath.

In feeding chambers, gel saliva structures of variable length were deposited during diet probing and remained attached to the lower side of Parafilm. Only salivary sheaths that adhered to the Parafilm over their full length were used for scanning electron microscopy. A typical salivary sheath produced under normal oxygen conditions (Fig. 2A, B) has a pearl-necklace like appearance [24] that narrows towards the tip and shows a number of short side-branches at regular distances. Each discrete “pearl” in the necklace is the result of secretion of a single drop of gel saliva. The sheath possesses a rough texture (Fig. 2B). This texture could represent a drying effect because sheaths dried on air for approximately 30 minutes. Drying and subsequent water absorption were observed for salivary sheaths (N = 5; Fig. S2).

Involvement of oxygen in sheath hardening – by formation of disulfide-bridges through oxidation of sulphydryl-groups was advanced by Miles [24] and Tjallingii [6]. This idea finds support in the appearance of sheaths produced under low-oxygen and reducing conditions. Feeding chambers that contained degassed diet and were loaded under Argon gas, are referred to as “low-oxygen”, rather than completely anoxic since Parafilm is permeable to air. The individual pearls of sheaths produced under low-oxygen conditions are not as discretely demarcated from one another as under regular oxygen conditions, presumably due to a slower hardening rate which allowed the individual drops of sheath saliva to merge before solidifying. Moreover, the entire sheath structure appears to be flattened and spread over a wider Parafilm surface, indicating that gel saliva remained in a fluid state for a longer time (Fig. 2C). 1 mM DTT at low-oxygen levels (Fig. 2D) makes the pearl-necklace structure barely recognizable. Small spherical nuclei inside the amorphous sheath may be interpreted as hardened saliva material that is beyond the influence of DTT.

### Inner structure of salivary sheaths

**Figure 3:**
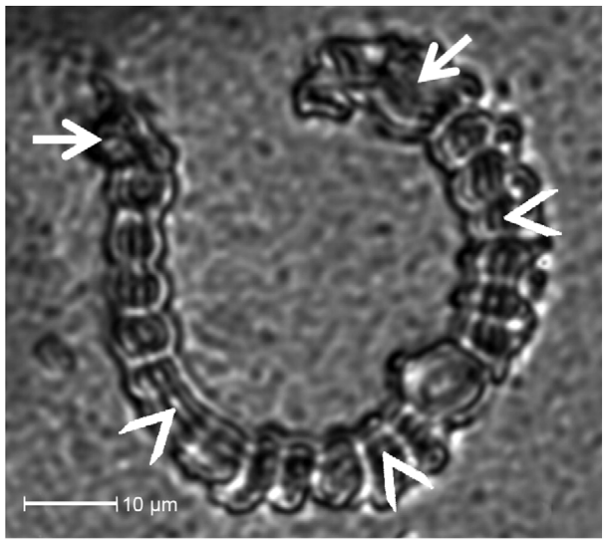
Optical sectioning by CLSM through a salivary sheath of *M. viciae*. The salivary sheath is attached along its whole length of 106 µm to the lower side of Parafilm membrane that covered a feeding chamber filled with ST diet. The site of stylet penetration through the Parafilm is located on the right end of the salivary sheath (right arrow). The sheath has a typical pearl-necklace like structure and narrows slightly towards the tip (left arrow). The empty stylet canal is visible inside the salivary sheath (indicated by arrowheads) with exception of the very first segment (right arrow).

After stylet retraction, an empty and clearly delimited tube-like stylet canal is left in the center of the salivary sheath (Fig. 3). Most sheath segments, individually hardened droplets of gel saliva, show a similar shape with exception of amorphous ones at penetration site and tip. Segments that appear to be branched have a globular appearance and a widened canal in relation to adjacent segments. Traces of a central canal are absent in the very first segment that is located at the penetration site.

### Collection of gel saliva proteins by degradation of salivary sheaths

A denaturating solution was developed to collect and separate proteins from salivary sheaths. The composition of the denaturating solution was based on the postulate that oxidation of sulphydryl-groups leads to sheath hardening [6], [24]. Urea, SDS and CHAPS in the solution were added with the intention to break hydrogen bonds and to increase solubility of proteins, while DTT was added to reduce sulphydryl groups and, hence, break disulphide bonds between proteins. Application of the solution on salivary sheaths led to severe disintegration of the sheath structure (Fig. S1A–C) and to complete solubilisation after approximately 75 min. Millipore water has no influence on the sheath structure (Fig. S1D–F). The experiment was repeated 5 times with identical results. Comparable results were obtained for salivary sheaths of *M. euphorbiae* whereat the salivary sheath solubilized completely after 40–60 min (data not shown).

### Protein composition of gel and watery saliva obtained with different diets

**Figure 4:**
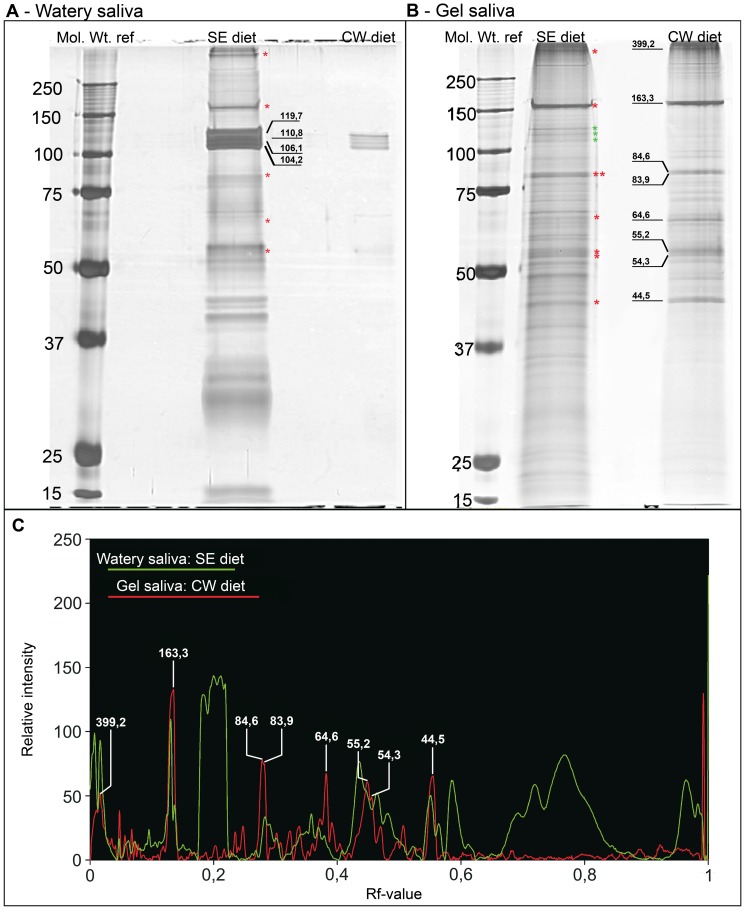
Separation and comparison of saliva protein fractions from *M. viciae* collected in SE – and CW-diet. Soluble and non-soluble protein fraction of saliva concentrate collected from SE-diet and CW-diet were separated in a 10% separation gel. Gels were silver stained. Lane 1 of each gel contains marker proteins Molecular weights (kDa) are given on the left. **A**) The soluble fraction from SE-diet shows 45 protein bands while in samples from CW-diet only 6 protein bands are detected. All latter protein bands match bands from the SE-diet but have a much lower abundance. **B**) Non-soluble fraction samples have fewer protein bands in CW-diet (lane 3, n = 66) than in SE-diet (lane 2, n = 87). Matching bands of lanes 2 and 3 show comparable intensities. The most intense protein bands (MWs are given in **B**) of the non-soluble fraction from CW-diet are used as reference proteins for this saliva fraction and are labeled with their MWs. Protein bands with corresponding MW in other lanes are marked with red asterisks. Protein bands of insoluble fraction that correspond to soluble fraction from SE-diet (**A**) are labeled with green asterisks and MWs are given in **A**. **C**) To discriminate protein bands of non-soluble fraction (red graph) from those of soluble fraction (green graph), soluble fraction from SE-diet (**A**, lane 2) was compared with non-soluble fraction from CW-diet (**B**, lane 3). The relative pixel intensity value (arbitrary units) is plotted against the retardation factor (Rf-value). Most intense proteins from non-soluble fraction (MWs are given), all of which have a relative pixel intensity >50, show a coincidence with 6 protein bands in the soluble fraction collected from SE-diet (red asterisks). Specific proteins from soluble fraction with a relative intensity of more than 50 (RF-value of approx. 0.2 (MWs in **A** and green asterisks in **B**) and higher 0.57), are only slightly visible in non-soluble fraction from CW-diet (**C**).

Samples of watery (soluble fraction) and gel saliva (non-soluble fraction), from SE- and CW-diet, respectively, were separated by 1D-SDS PAGE. The experiment was repeated 3 times with identical results. Protein compositions of the fractions were compared to determine the impact of the two diets on saliva secretion (Fig. 4A, B). The soluble fraction from SE-diet contains 45 protein bands and from CW-diet only 6 protein bands. The non-soluble fraction from SE-diet contains 87 protein bands while that from CW-diet contained approximately 65 protein bands. Furthermore, relative band intensities were plotted for soluble fraction from SE-diet and for non-soluble fraction from CW-diet to allow a better comparison of respective fraction types with each other (Fig. 4C). These samples were chosen for direct comparison of soluble and non-soluble fraction because i) secretion of the soluble fraction mainly occurs in SE-diet (Fig. 4A) and in consequence ii) the non-soluble fraction from CW-diet was assumed to contain no contamination of soluble fraction since secretion of latter is nearly absent (Fig. 4B vs. Fig. 4A). For detailed comparison, the most prominent protein bands of the insoluble fraction with MWs of 44,5; 54,3; 55,2; 64,6; 83,9; 84,6; 163,3 and 399,2 kDa (relative pixel intensity value of >50, Fig. 4C) were selected from CW-diet sample as a reference (Fig. 4B) and are labeled with red asterisks when present in other samples (Fig. 4A, B).

The most intense protein bands from SE-diet (104,2; 106,1; 110,8 and 119,7 kDa) were detected merely at low intensities in soluble fraction from the CW-diet. In addition, similar protein bands were detected in insoluble fraction from SE-diet (Fig. 4B, green asterisks) and are absent in insoluble-fraction samples from CW-diet.

### Reducing abilities of watery saliva

Reducing abilities of watery-saliva proteins from *M. viciae* were tested (N = 2) by mixing watery-saliva samples with methylene blue. The dye is dark blue when in its oxidized state and becomes colorless if exposed to a reducing agent.

After 72 h that methylene blue was reduced by watery saliva, indicated by a color change of the solution from dark to bright blue. The extinction values for methylene blue exposed to watery saliva proteins were 1.705 and 1.066 in the two replicates. Extinction values of methylene blue mixed with water (control) were 2.693 and 2.719. A reduction of extinction by watery saliva samples of 46,7% and 60,8% was measured. The solution surface of the upper solution in the reaction tube, which was plugged but not filled completely, was dark blue, indicating a reoxidation of the methylene blue by oxygen in the space in the tube above the solution.

## Discussion

### The salivary flange and the stylet piercing site

Gel saliva secretion starts with the formation of a salivary flange on the surface of the plant. The salivary flange has been suggested to prevent the stylet from slipping sideways while the epidermal surface is being pierced [17]. It appears that the entry of the stylet canal is sealed after stylet retraction because no penetration hole in the salivary flange is visible (Fig. 3C,D) and no stylet canal can be detected in the first segments of salivary sheaths where stylets were retracted (Fig. 3). The plug material likely consists of gel saliva given its appearance in SEM (Fig. 1). A potential reason for stylet canal filling with gel saliva could be a facilitated withdrawal of the stylet from the sheath by repulsion. This would eliminate possible suction effects inside the thin stylet canal of the salivary sheath.

### The salivary sheath secretion process

The salivary sheath is formed during stylet propagation inside the plant and envelops the stylet. In an artificial environment, the sheath possesses a pearl-necklace like structure (Figs. 2 and 3), which was not observed for stylet sheaths extracted from plant tissue [2]. The absence of bulging may be due to shaping by the stylet environment inside the plant. The pearl-necklace structure is formed by two saliva secretion processes [22]. An initial secretion of a droplet of gel saliva is followed by secretion of a small volume of watery saliva that serves to inflate the droplet to a spherical structure, creating a spherical cavity inside the droplet of gel saliva. The distinct tubular structure inside the salivary sheath observed by CLSM (Fig. 3), conflicts with this proposition and indicates that all saliva material hardens after being pierced by the stylet. Probably, the “watery saliva” inflating the droplets of gel saliva [22] was actually gel saliva in its initially secreted liquid state that shows a delayed hardening due to less contact with oxygen. Thus, salivary sheaths appear to be formed only by successive pulses of gel saliva as is supported by the almost exclusive secretion of gel saliva under cell-wall conditions (Fig. 4A).

### Formation and solidification of the salivary sheath

Gel saliva has been assumed to harden by oxidation of sulphydryl-groups [6], [24]. In keeping with this idea, the formation of saliva deposits with less distinctive droplets under low-oxygen conditions indicates an involvement of oxygen in the hardening process (Fig. 2C). Furthermore, defective sheaths were found in diets containing the strongly reductive agent DTT [31]. DTT reduces disulfide bonds and prevents their intramolecular and intermolecular formation. In the presence of DTT, sheath formation was incomplete with vague, spherical structures in the sheath center, possibly due to limited diffusion of DTT into the sheath material (Fig. 2D).

A protein (ACYPI009881) with a predicted molecular mass of 130 kDa and high cysteine content was identified in *A. pisum* saliva and is a potential candidate as a structural sheath protein [9]. Sequences with a close similarity are present in *M. persicae* EST database (EST accessions EC387934, EC388457 and EE572212 in NCBI). ACYPI009881 is most likely not specific to the pea aphid [9], consistent with its putative role as a major sheath protein and the presence of a salivary sheath in all aphid species studied so far. Protein bands of a MW similar to the sheath protein were detected in the soluble saliva fraction from D*iuraphis noxia, D. tritici, D. mexicana,* and S*chizaphis.graminum* [32]. Formation of a sheath-like structure was also observed for other groups of the Sternorrhyncha such as whiteflies [33] and for planthoppers (Fulgoromorpha) [34]. As a speculation, the two sister groups Sternorryhncha and Fulgoromorpha (cf. the phylogenetic tree by [35]) could possess a possible overlap of -protein sequence of the 130 kDa sheath protein that originates from a common ancestor.

### Dissolving salivary sheaths

To enable biochemical studies on gel-saliva proteins, salivary sheaths were collected by a novel sheath-degradation solution (Fig. S1), which was designed on the basis of the putative presence of disulfide bonds in sheath material [9], [24] and their assumed function in sheath hardening [6]. Subsequent preparation enabled a high-resolution separation using 1D-SDS-PAGE (Fig. 4) which may be useful for functional and proteomic studies of gel-saliva components from other plant-sucking insects as well.

### Secretion of watery and gel saliva under different conditions

High amounts of watery saliva (soluble fraction) were secreted into SE-diet (pH 7.2) as observed before (e.g. [7]), while only the six most intense protein bands from soluble fraction in SE-diet were detected under CW-simulating conditions (pH 5.0) (Fig. 4A). By implication, watery saliva proteins will hardly be secreted under CW-conditions (Fig. 4B) and protein bands detected in Parafilm deposits must be gel-saliva (non-soluble fraction) components. It was puzzling, that the number of protein bands from non-soluble deposits on Parafilm was higher under SE-conditions (Fig. 4B, lane 2) than under CW-conditions (Fig. 4B, lane 3). However, the higher number of proteins deposited under SE-conditions may be attributed to watery-saliva proteins, because protein bands of the non-soluble fraction merely represent gel-saliva proteins under CW-conditions (watery saliva proteins are virtually absent, Fig. 4A, lane 3). Components of the soluble protein fraction may be incorporated into non-polymerized sheath material. In turn, leaching of non-soluble fraction components can be induced by retarded hardening of sheath structure in the diet environment which is corroborated by the reducing abilities of watery saliva. Another possibility could be that watery saliva and gel saliva show some overlap in their protein composition because of a common basic protein composition, while secretion of only a couple of proteins is varied; specific proteins are added or removed from saliva or their quantity is changed, so that the type of saliva changes.

We tried to identify protein bands specific to the saliva types by comparing the protein patterns of the soluble fraction from the ST-diet and the non-soluble fraction from the CW-diet (Fig. 4C). Proteins with a MW of 100–120 kDa, shown to be responsible for calcium-binding [7], appear to be attributed to the soluble fraction (red asterisks, Fig. 4A). Another group of proteins that are only present in the soluble fraction have MWs of around 30 kDa (Fig. 4). There are functional indications that the sheath protein [9] and cell-wall degrading enzymes (e.g. [18]) are part of the non-soluble fraction, but it is difficult to attribute specific protein bands to the non-soluble fraction since the soluble fraction contains bands with similar MWs (black asterisks, Fig. 4).

### Diet composition influences the type of secreted saliva

Watery saliva (soluble fraction) is almost exclusively secreted (Fig. 4A) under ST-conditions and gel saliva (non-soluble fraction) preferably under CW-conditions (Fig. 4). Secretion of watery saliva has been associated with pH, sucrose concentration, and the presence of amino acids or a combination of these factors [1]. pH 7 and sucrose concentrations of approx. 400 mM, which are in the range of the SE-content, provide the most favorable conditions for secretion of watery saliva as indicated by electrical penetration graph recordings [1].

The adaptive secretion of salivary fractions (Fig. 4) indicates that aphids are able to sense their stylet milieu and secrete saliva components appropriate for the detected conditions. Therefore, aphids must permanently take up plant fluids, not only from the cell lumina [3], but also from the apoplast. The physiological impact is obvious: aphids secrete a type of saliva, as a part of a flexible behavior, which is adapted best to the foraging requirements. Since an aphid stylet lacks chemosensillae, fluid must reach the precibarial chemosensilla [36], which are located in the nutrition channel before the precibarial valve [37].

## Supporting Information

Figure S1 **Solubilisation of salivary sheaths from** ***M. viciae*** **by denaturating solution. S**alivary sheaths, attached to the lower side of Parafilm®, were observed with bright field microscopy (40× water immersion objective). Images were taken at t = 0 min (**A, D**), t = 20 min (**B, E**) and t = 40 min (**C, F**) after application of the respective solvent (denaturating solution [**A–C**] and water [**D–F**]). Denaturating solution shows after 20 min (**B**) a swelling of the sheath structure in the region of the tip and a stronger bending of this area, indicating a detachment of this part of the sheath from the Parafilm®. The shape of the salivary sheath appears less structured, indicating a beginning disintegration of the structure. After 40 min (**C**) the sheath structure becomes less differentiated against the surrounding whereas the outer shape is still observable. Water as control solvent has no observable influence on the sheath structure during the observation period (**D–F**).
(TIF)

Figure S2 **Absorption of water by salivary sheath of** ***M. viciae*** **. S**alivary sheaths on Parafilm®, were observed with bright field microscopy (40× water immersion objective). Images were taken at t = 0 s (**A**) and t = 30 s (**B**). A salivary sheath that dried for approximately 30 minutes (**A**) swells after addition of water and reaches its final size within a few seconds (**B**).
(TIF)

## References

[1] Hewer A, Will T, van Bel AJE (2010) Plant cues for aphid navigation in vascular tissues. J Exp Biol 213: 4030–4042. 21075945 10.1242/jeb.046326

[2] Hewer A, Becker A, van Bel AJE (2011) An aphid's Odyssey. The cortical quest for the vascular bundle. J Exp Biol 214: 3868–3879. 22031752 10.1242/jeb.060913

[3] Martin B, Collar JL, Tjallingii WF, Fereres A (1997) Intracellular ingestion and salivation by aphids may cause the acquisition and inoculation of non-persistently transmitted plant viruses. J Gen Virol 78: 2701–2705. 9349493 10.1099/0022-1317-78-10-2701

[4] Powell G (2005) Intracellular salivation is the aphid activity associated with inoculation of non-persistently transmitted viruses. J Gen Virol 86: 469–472. 15659767 10.1099/vir.0.80632-0

[5] Ponsen MB (1972) The site of potato leafroll virus multiplication in its vector, *Myzus persicae* . Mededelingen van de Landbouwhogeschool Wageningen 16: 1–147.

[6] Tjallingii WF (2006) Salivary secretions by aphids interacting with proteins of phloem wound responses. J Exp Bot 57: 739–745. 16467410 10.1093/jxb/erj088

[7] Will T, Tjallingii WF, Thönnessen A, van Bel AJE (2007) Molecular sabotage of plant defense by aphid saliva. Proc Natl Acad Sci U S A 104: 10536–10541. 17553961 10.1073/pnas.0703535104 PMC1965548

[8] Mutti NS, Louis J, Pappan LK, Pappan K, Begum K, et al (2008) A protein from the salivary glands of the pea aphid, *Acyrthosiphon pisum*, is essential in feeding on a host plant. Proc Natl Acad Sci U S A 105: 9965–9969. 18621720 10.1073/pnas.0708958105 PMC2481341

[9] Carolan JC, Fitzroy CIJ, Ashton PD, Douglas AE, Wilkinson TL (2009) The secreted salivary proteome of the pea aphid *Acyrthosiphon pisum* characterised by mass spectrometry. Proteomics 9: 2457–2467. 19402045 10.1002/pmic.200800692

[10] Knoblauch M, van Bel AJE (1998) Sieve tubes in action. Plant Cell 10: 35–50.

[11] Will T, van Bel AJE (2006) Physical and chemical interactions between aphids and plants. J Exp Bot 57: 729–737. 16473888 10.1093/jxb/erj089

[12] Furch ACU, Hafke JB, Schulz A, van Bel AJE (2007) Ca^2+^-mediated remote control of reversible sieve tube occlusion in *Vicia faba* . J Exp Bot 58: 2827–2838. 17615409 10.1093/jxb/erm143

[13] Furch ACU, van Bel AJE, Fricker MD, Felle HH, Fuchs M, et al (2009) Sieve element Ca^2+^-Channels as relay stations between remote stimuli and sieve tube occlusion in *Vicia faba* . Plant Cell 21: 2118–2132. 19602624 10.1105/tpc.108.063107 PMC2729599

[14] Furch ACU, Zimmermann MR, Will T, Hafke JB, van Bel AJE (2010) Remote-controlled stop of phloem mass flow by biphasic occlusion in *Cucurbita maxima* . J Exp Bot 13: 3697–3708. 10.1093/jxb/erq181 PMC2921205 20584788

[15] Will T, Kornemann SR, Furch ACU, Tjallingii WF, van Bel AJE (2009) Aphid watery saliva counteracts sieve-tube occlusion: a universal phenomenon? J Exp Biol 212: 3305–3312. 19801435 10.1242/jeb.028514

[16] Bos JIB, Prince D, Pitino M, Maffei ME, Win J, et al (2010) A Functional genomics approach identifies candidate effectors from the aphid species *Myzus persicae* (green peach aphid). PLoS Genetics 6: e1001216. 21124944 10.1371/journal.pgen.1001216 PMC2987835

[17] Miles PW (1999) Aphid saliva. Biol Rev 74: 41–85.

[18] Cherqui A, Tjallingii WF (2000) Salivary proteins of aphids, a pilot study on identification, separation and immunolocalisation. J Insect Physiol 46: 1177–1186. 10818245 10.1016/s0022-1910(00)00037-8

[19] Pollard DG (1973) Plant penetration by feeding aphids (Hemiptera: Aphidoidea): a review. Bull Entomol Res 62: 631–714.

[20] Tjallingii WF, Hogen Esch Th (1993) Fine structure of aphid stylet routes in plant tissues in correlation with EPG signals. Physiol Entomol 18: 317–328.

[21] Kimmins FM (1986) Ultrastructure of the stylet pathway of *Brevicoryne brassicae* in host plant tissue, *Brassica oleracea* . Ent Exp App 41: 283–290.

[22] McLean DL, Kinsey MG (1965) Identification of electrically recorded curve patterns associated with aphid salivation and ingestion. Nature 205: 1130–1131. 5833223 10.1038/2051130a0

[23] Miles PW, McLean DL, Kinsey MG (1964) Evidence that two species of aphid ingest food through an open stylet sheath. Experientia 20: 582. 10.1007/BF02150309 5859228

[24] Miles PW (1965) Studies on the salivary physiology of plant-bugs: the salivary secretions of aphids. J Insect Physiol 11: 1261–1268. 5828294 10.1016/0022-1910(65)90119-8

[25] Miles PW (1987) Plant-sucking bugs can remove the contents of cells without mechanical damage. Experientia 43: 937–939.

[26] van der Westhuizen AJ, Qian X-M, Botha A-M (1998) Differential induction of apoplastic peroxidase and chitinase activities in susceptible and resistant wheat cultivars by Russian wheat aphid infestation. Plant Cell Rep 18: 132–137.

[27] Cosgrove DJ, Cleland RE (1983) Solutes in the free space of growing stem tissues. Plant Physiol 72: 326–331. 16663001 10.1104/pp.72.2.326 PMC1066232

[28] Laemmli UK (1970) Cleavage of structural proteins during the assembly of the head of bacteriophage T4. Nature 227: 680–685. 5432063 10.1038/227680a0

[29] Schoenle EJ, Adams LD, Sammons DW (1984) Insulin-induced rapid decrease of a major protein in fat cell plasma membranes. J Biol Chem 259: 12112–12116. 6434536

[30] Switzer RC 3rd, Merril CR, Shifrin S (1979) A highly sensitive silver stain for detecting proteins and peptides in polyacrylamide gels. Anal Biochem 98: 231–237. 94518 10.1016/0003-2697(79)90732-2

[31] Cleland WW (1964) Dithiothreitol, a new protective reagent for SH groups. Biochemistry 3: 480–482. 14192894 10.1021/bi00892a002

[32] Cooper WR, Dillwith JW, Puterka GJ (2011) Comparison of salivary proteins from five aphid (Hemiptera: Aphididae) species. Environ Entomol 40: 151–156. 22182624 10.1603/EN10153

[33] Freeman TP, Buckner JS, Nelson DR, Chu Chang C-C, Henneberry TJ (2001) Stylet penetration by *Bemisia argentifolii* (Homoptera: Aleyrodidae) into host leaf tissue. Annals of the Entomological Society of America 94: 761–768.

[34] Brentassi ME, Remes Lenicov AMM de (2007) Feeding behavior of the vector *Delphacodes kuscheli* (Hemiptera: Fulgoromorpha: Delphacidae) on maize and oat. Ann Soc Entomol Fr 43: 205–212.

[35] Song N, Liang A-P (2009) Complete mitochondrial genome of the small brown planthopper, *Laodelphax striatellus* (Delphacidae: Hemiptera), with a novel gene order. Zoolog Sci 26: 851–860. 19968473 10.2108/zsj.26.851

[36] Wensler RJ, Filshie BK (1969) Gustatory sense organs in the food canal of aphids. J Morphol 129: 473–92.

[37] McLean DL, Kinsey MG (1984) The precibarial valve and its role in the feeding behavior of the pea aphid, *Acyrthosiphon pisum* . Bulletin of the Entomological Society of America 30: 26–31.

