# Supplementary figures and images for: Aphid Gel Saliva: Sheath Structure, Protein Composition and Secretory Dependence on Stylet-Tip Milieu
Source: PLoS One. 2012 Oct 2;7(10):e46903. doi: 10.1371/journal.pone.0046903 (PMC3462764; doi:10.1371/journal.pone.0046903)

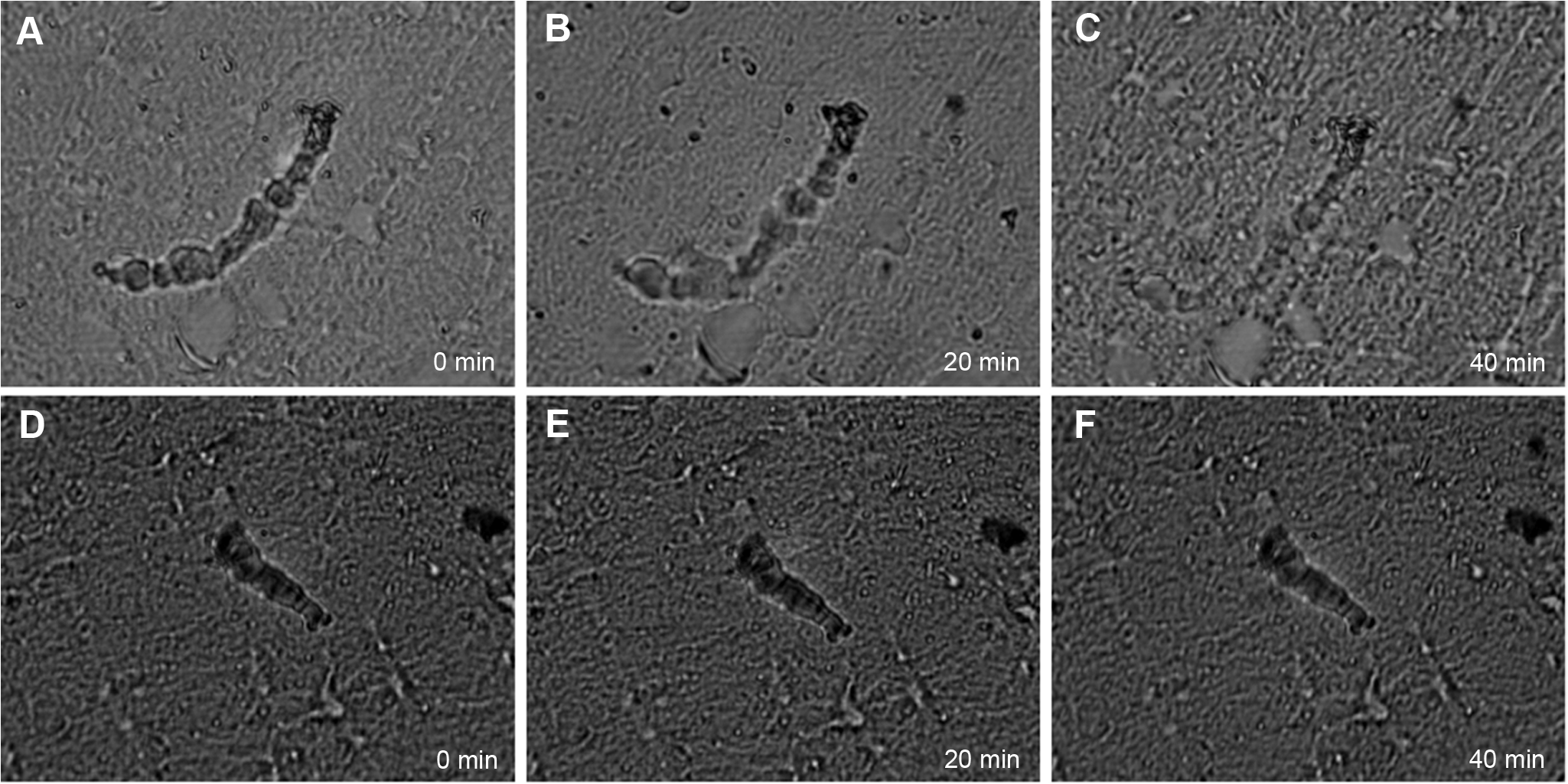

Supplement: Figure S1 — Solubilisation of salivary sheaths from M. viciae by denaturating solution. Salivary sheaths, attached to the lower side of Parafilm®, were observed with bright field microscopy (40× water immersion objective). Images were taken at t = 0 min (A, D), t = 20 min (B, E) and t = 40 min (C, F) after application of the respective solvent (denaturating solution [A–C] and water [D–F]). Denaturating solution shows after 20 min (B) a swelling of the sheath structure in the region of the tip and a stronger bending of this area, indicating a detachment of this part of the sheath from the Parafilm®. The shape of the salivary sheath appears less structured, indicating a beginning disintegration of the structure. After 40 min (C) the sheath structure becomes less differentiated against the surrounding whereas the outer shape is still observable. Water as control solvent has no observable influence on the sheath structure during the observation period (D–F). (TIF) [file pone.0046903.s001.tif]

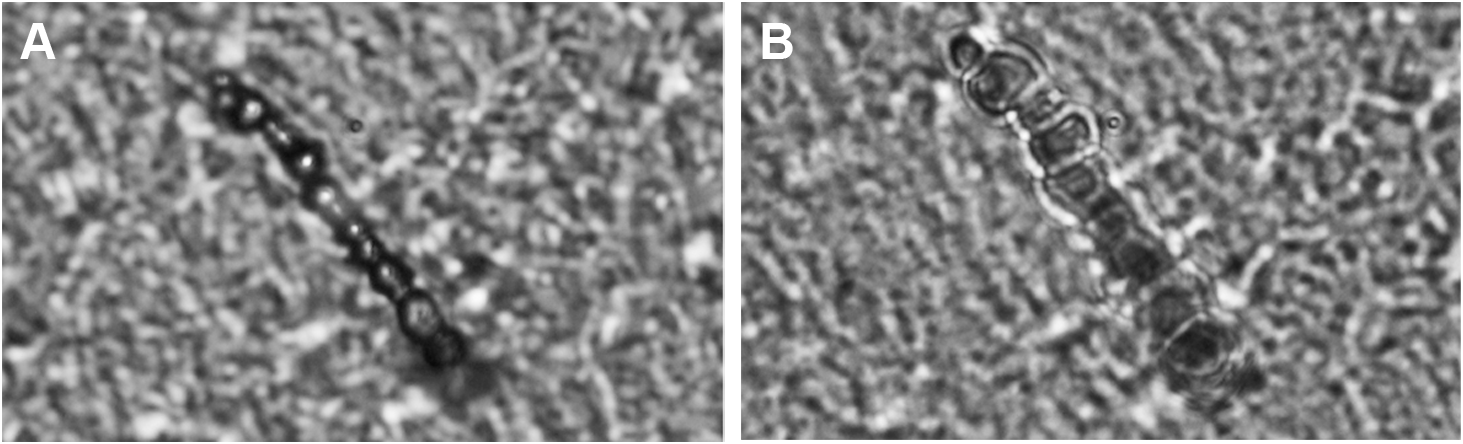

Supplement: Figure S2 — Absorption of water by salivary sheath of M. viciae . Salivary sheaths on Parafilm®, were observed with bright field microscopy (40× water immersion objective). Images were taken at t = 0 s (A) and t = 30 s (B). A salivary sheath that dried for approximately 30 minutes (A) swells after addition of water and reaches its final size within a few seconds (B). (TIF) [file pone.0046903.s002.tif]
